# Long non-coding RNA growth arrest specific transcript 5 acting as a sponge of MicroRNA-188-5p to regulate SMAD family member 2 expression promotes myocardial ischemia-reperfusion injury

**DOI:** 10.1080/21655979.2021.1957524

**Published:** 2021-10-10

**Authors:** Jin Xu, Dong Yu, Xiaolu Bai, Peng Zhang

**Affiliations:** a Department of Anesthesiology, Shanghai East Hospital, Tongji University School of Medicine, Shanghai City, China; b Department of Cardiology, Minhang Hospital of Zhongshan Hospital Affiliated to Fudan University (Central Hospital, Minhang District, Shanghai), Shanghai City, China

**Keywords:** Long non-coding RNA growth arrest specific transcript 5, microRNA-188-p, smad2, myocardial ischemia-reperfusion injury, NF-ΚB

## Abstract

The purpose of this work is to probe into the potential role of long non-coding RNA growth arrest specific transcript 5 (lncGAS5)/ microRNA (miR)-188-5p/SMAD2 axis in MIRI. Through ligating the left anterior descending (LAD) coronary artery, MIRI animal model and hypoxia/reoxygenation (H/R) myocardial injury model *in vitro* were established. Via adenovirus or plasmid transfection, lncGAS5/MiR-188-5p/SMAD2 expression was up-regulated or down-regulated in the study. RT-qPCR was applied to check LncGAS5/MiR-188-5p/SMAD2 mRNA expression, HE staining for histopathological staining, TUNEL staining and flow cytometry to examine cardiomyocyte apoptotic rate, CCK-8 to check cell viability, ELISA to detect inflammatory factor levels, Western blot to examine Bax, Bcl-2, cleaved caspase-3, NF-κB and SMAD2 expression, and dual luciferase reporter experiment to examine the targeting relationship of miR-188-5p with LncGAS5 and SMAD2. The results indicated that LncGAS5 and SMAD2 were highly expressed in MIRI and miR-188-5p was under-expressed. Silencing LncGAS5 and SMAD2 or overexpressing miR-188-5p could reduce MIRI in myocardial tissue, cardiomyocyte apoptosis, inhibit Bax, cleaved caspase-3 and NF-κB expressions and promote Bcl-2 expression, while reducing inflammatory factors TNF -α, IL-1β and IL-6 levels. Overexpressing LncGAS5 promoted MIRI. Additionally, the impact of silencing LncGAS5 on MIRI could be reversed through inhibiting miR-188-5p. LncGAS5 acted as a sponge of miR-188-5p to target SMAD2 expression. In conclusion, Silencing LncGAS5 is available to improve MIRI through regulating miR-188-5p/SMAD2 axis, and may be used as a potential target for treating MIRI in the future.

## 1 Introduction

Acute myocardial infarction (AMI) resulting from insufficient blood flow to the heart, features cardiac ischemia and hypoxia [1]. AMI has become one of the leading causes of death worldwide [2]. Currently, the most effective way to save AMI is to perform mechanical revascularization [3]. But amid revascularization, myocardial ischemia reperfusion injury (MIRI) will inevitably appear, leading to increased pro-inflammatory factors and cardiomyocyte apoptosis [4,5]. Therefore, saving pro-inflammatory programmed cardiomyocyte apoptosis has become a research hotspot for improving MIRI.

Long non-coding RNA (LncRNA), an essential subject in the non-coding RNA family, exists in large numbers in tissues and cells and has been shown to be involved in many vital regulatory processes like chromatin modification, transcription activation, and transcription interference [6]. Based on recent studies, LncRNA is involved in regulating MIRI cardiomyocyte apoptosis, and this phenomenon has been confirmed in some specific experiments. For example, Lou Z et al. found that there are 1395 LncRNA differential expressions in human ischemia-reperfusion myocardial tissue through LncRNA microarray analysis [7]. A recent research reported that LncRNA TUG1 competitively binds to miR-340, thereby accelerating MIRI [8]. Additionally, studies have revealed that lncRNA ROR/miR-124-3p/TRAF6 axis regulation acts pivotally in MIRI-induced inflammatory response in human cardiomyocytes [9]. Therefore, in-depth exploration of the role of lncRNA in MIRI has far-reaching significance for understanding MIRI pathogenesis and treatment. Long non-coding RNA growth arrest specific transcript 5 (lncGAS5) is important for LncRNA family, and it has been found to act pivotally in regulating cancer, rhinitis, and systemic lupus erythematosus [10–12]. Recent studies have found that lncGAS5 is differentially expressed in MIRI models, and that lncGAS5 has a promoting impact on MIRI cardiomyocyte apoptosis [13,14]. But the underlying molecular mechanism of lncGAS5 affecting MIRI has not been fully elucidated.

In this research, the author explored the potential molecular mechanism of lncGAS5 affecting MIRI through changing lncGAS5 expression in MIRI mice models and hypoxia/reoxygenation (H/R) *in vitro* models, focusing on exploring the downstream target of lncGAS5 in MIRI model and its influence on apoptosis-related proteins Bax, Bcl-2, cleaved caspase-3 and NF-κB signaling pathway.

## 2 Methods

### 2.1 Ethic standard and informed consent in patients

Clinical trials were conducted in line with the declaration of the World Medical Association. The research scheme was approved by the Ethics Committee of Minhang Hospital of Zhongshan Hospital Affiliated to Fudan University. All subjects were given written informed consent prior to inclusion in the study after being fully informed of the purpose, nature of and risks of participation.

### 2.2 Inclusion and exclusion criteria for patients with AMI

Twenty-six patients with AMI were enrolled with ST-segment elevation caused only mainly by the left anterior descending coronary artery (LAD). According to the guidelines of the American College of Cardiology/American Heart Association on coronary angiography and PCI, the primary percutaneous coronary intervention (PCI) is performed by an experienced interventionist within 12 h of the onset of chest pain. Blood samples were collected 48 h before and after PCI and lncGAS5 expression was measured by reverse transcription quantitative polymerase chain reaction (RT-qPCR) [15].

Patients with 2 or more of the following conditions were included in the study:(1) chest pain; (2) Electrocardiograph changes with Q wave/ST segment elevation; (3) Up-regulation of serum creatine kinase-myoglobin fraction/troponin I (cTnI).

Exclusion criteria were as follows: (1) cardiogenic shock; (2) left main coronary artery occlusion or severe stenosis; (3) Infarction-related arterial blood flow ≥ thrombolysis in grade 1 myocardial infarction; (4) Clenbuterol level ≥1 indicated apparent coronary collateral in the risk area; (5) Infection or surgery occurred within 2 weeks.

### 2.3 MIRI model establishment and grouping

Specific pathogen-free male c57BL6/J mice (20–24 g, Minhang Hospital, Zhongshan Hospital Affiliated to Fudan University Animal Experiment Research Center) were placed in a standard, pathogen-free environment, with constant temperature (25°C ± 2°C) and humidity (50%-60%). An animal model of MIRI was established as described above [16]. All animals were allowed to eat and drink pure water freely, and the MIRI model was established through ligating LAD coronary artery. In short, after anesthetizing mice with 3% sodium pentobarbital (Sigma, Shanghai), longitudinal incision was performed on the left side of the sternum to expose 4^th^ rib, ligated at about 3 mm away from LAD coronary artery origin with a 6–0 thread, later squeezed out the gas and sutured. The mice in the Sham group were only threaded and not ligated. As shown in Figure S1, after 30 min, the knot was loosened and reperfusion was performed. Aiming to silence LncGAS5, during I/R modeling process, 5 × 10^12^ vg/mL adenovirus vector was injected into the left ventricle, and the aorta was clamped for 10 s. In accordance with heart contraction, the virus was transfected into heart muscle through coronary arteries, Sh-LncGAS5 and sh-NC adenovirus vectors were designed and synthesized via Obio Technology (China). all procedures were carried out in accordance with the Guide to the Care and Use of Experimental Animals issued by China Laboratory Animal Care Association and approved by the Animal Research Ethics Committee of Minhang Hospital, Zhongshan Hospital Affiliated to Fudan University.

### 2.4 TdT-mediated dUTP-biotin nick end-labeling (TUNEL) for cell apoptosis detection

In line with the instructions, TUNEL cell apoptosis detection kit (Shanghai Biotech Research Institute, Shanghai) was applied to assess cardiomyocyte apoptosis. In short, myocardial tissue was deparaffinized in xylene, dehydrated with graded ethanol, and later incubated with 100 mg/ml proteinase K in a wet box at 37°C for 30 min. These sections later were incubated with TUNEL reaction mixture at 37°C for 1 h and visualized via transcriptional activator protein with nickel-diaminobenzidine. The apoptosis rate was the ratio of apoptotic cells to total cells per unit area.

### 2.5 Hematoxylin-eosin (HE) staining

The myocardial tissue was fixed in 4% paraformaldehyde for 24 h, and later in paraffin cut into continuous coronal sections (4 µm), which were deparaffinized in xylene and later hydrated in gradient ethanol. HE staining was applied to observe myocardial tissue and images were taken via microscope digital camera system (DP12 SZX7, Olympus, Japan).

### 2.6 Cell culture and H/R treatment

The H/R model was established by referring to the previous method [17]. HL-1 cells from the Chinese Academy of Sciences (Shanghai) were cultured in Dulbecco’s Modified Eagle Medium (DMEM) (Gibco-BRL, Grand Island, NY) at 37°C with 5% CO_2_, and seeded in the logarithmic growth phase into 24-well plates. Amid H/R modeling, under hypoxic condition, a mixture of 95% N_2_ and 5% CO_2_ were added for 6 h, and later the medium was discarded, and added with fresh DMEM to reoxygenate 24-well plates, and untreated cardiomyocytes were used as the blank control group.

### 2.7 Cell transfection

oe-LncGAS5, oe-negative control (NC), oe-SMAD2, si-SMAD2, si-LncGAS5 and si-NC were designed and synthesized by Obio Technology (China). miR-188-5p mimic/inhibitor and corresponding NC were provided by VipotionBio Co., Ltd. (China). After being cultured overnight, the cells were transfected with the above plasmids or oligonucleotides via Lipofectamine 2000 (Invitrogen, U.S.). Twelve hours after transfection, the cardiomyocytes were treated with hypoxia (24 h) and reoxygenation (12 h).

### 2.8 Cell counting kit (CCK)-8 assay for cell viability detection

After seeding the cardiomyocytes into 96-well plates, 100 μL of medium was added to each well for culture, overnight at 37°C and 5% CO_2_. After 12-h transfection, hypoxia was performed for 24 h and reoxygenation for 12 h. After that, 10 μL of CCK-8 solution (Dojindo Laboratories, Japan) was added to each well, and incubated for 1–2 h at 37°C, and the optical density (OD) value was later measured in each well at 450 nm via ELIASA.

### 2.9 Flow cytometry for apoptosis detection

Via Fluoresceinisothiocyanat (FITC) Annexin V/apoptosis kit (Thermo Fisher Scientific), the cell apoptosis was measured, and the cardiomyocytes were stained with Annexin V-FITC and propidium iodide (Thermo Fisher Scientific) for 15 min in the darkness. Subsequently, the percentage of apoptotic cells was analyzed on a flow cytometer (FACScan; BD Biosciences, U.S.) via CellQuest software (BD Biosciences).

### 2.10 Enzyme-linked immunosorbent assay (ELISA)

Based on the kit instructions, IL-1β, IL-6 and TNF-α ELISA kits (Hangzhou MultiScience Biotech Co., Ltd) were applied to measure corresponding pro-inflammatory cytokine expressions in myocardial tissue and cells, as well as Spectramax i3 multi-mode detection platform (Molecular Devices, U.S.) to record the absorbance at 450 nm.

### 2.11 RT-qPCR

Total RNA was extracted from myocardial tissues and cells via TRIzol reagent (Invitrogen, U.S.), and cDNA was synthesized with RT-qPCR kit (Bestar; DBI Bioscience). Through Green PCR Master Mix (DBI Bioscience), RT-qPCR was performed in Stratagene RT-PCR system (Applied Biosystems, U.S.). Through 2^−ΔΔCt^, the relative gene expression was quantified. Glyceraldehyde-3-phosphate dehydrogenase (GAPDH) and U6 were used as internal controls, and the primer sequences were shown in Table 1.

**Table 1.** RT-qPCR primer sequence

	Primer sequence (5′ – 3′)
GAPDH	Forward: 5ʹ-CCTCGTCTCATAGACAAGATGGT-3’
	Reverse: 5ʹ-GGGTAGAGTCATACTGGAACATG-3’
U6	Forward: 5ʹ-CTCGCTTCGGCAGCACA-3’
	Reverse: 5ʹ-AACGCTTCACGAATTTGCGT-3’
LncGAS5	Forward: 5ʹ- TGGTTCTGCTCCTGGTAACG −3’
	Reverse: 5ʹ- AGGATAACAGGTCTGCCTGC-3’
MiR-188-5p	Forward: 5ʹ- CATCCCTTGCATGGTGGAG-3’
	Reverse: 5ʹ- CTCAACTGGTGTCGTGGAGTC-3’
SMAD2	Forward: 5ʹ- TACCCACTCCATTCCA-3’
	Reverse: 5ʹ- TGATAAACGGCCTCAA-3’

Note: GAPDH, Glyceraldehyde-3-phosphate dehydrogenase; LncGAS5, long non-coding RNA growth arrest specific transcript 5; MiR-188-5p, microRNA-188-5p.

### 2.12 Western blot

The tissues and cells were lysed via Radio-Immunoprecipitation assay lysis buffer (Beyotime, China), and the total protein concentration was measured via Pierce bicinchoninic acid (BCA) protein analysis kit (Pierce, Rockford). After elution in sample loading buffer (Beyotime) and separation via sulfate polyacrylamide gel electropheresis (SDS-PAGE), the sample was transferred to Polyvinylidene fluoride (PVDF) membranes at a constant current of 200 mA, which was blocked with 5% skimmed milk in Tris-buffered saline Tween (TBST) at room temperature, incubated with primary antibody GAPDH (2118, Cell Signaling Technology), SMAD2 (5339, Cell Signaling Technology), Bax (50,599-2- Ig, Proteintech), Bcl-2 (sc-7382, Santa Cruz Biotechnology), Cleaved caspase-3 (9664, Cell Signaling Technology), Caspase-3 (19,677-1-AP, Proteintech), NF-κB p65 (8242, Cell Signaling Technology), and p-NF-κB p65 (3031, Cell Signaling Technology). After washing in triplicate with Tris-buffered saline with Tween 20 (TBST), the membrane was incubated with the secondary antibody (goat anti-rabbit Immunoglobulin G (IgG), ab6721) in incubators for 1.5 h at room temperature, washed again with TBST 3 times. The membrane was treated with electrogenerated chemiluminescence (ECL) Plus substrate from Life Technologies Corporation (Gaithersburg, U.S.) to detect protein signals, and the image acquisition and analysis system of Lab Works 4.5 software (SIL Technologies, U.S.) were applied to detect and quantify the immunoreactive signal of protein bands.

### 2.13 Dual luciferase reporter assay

WT-LncGAS5, MUT-GAS5, WT-SMAD2 as well as MUT-SMDA2 fragments were cloned into pmirGLO luciferase reporter vector (Promega, U.S.), and later Lipofectamine 2000 was applied to co-transfect the above-mentioned vectors with miR-188-5p mimic or mimic-NC into cardiomyocytes. After 48 h, via dual luciferase reporter gene assay system (Promega), the luciferase activity was detected with Renilla luciferase activity as the endogenous control.

### 2.14 Data analysis

The experimental findings were expressed as mean ± standard deviation (SD), and SPSS 22 software was applied for data analysis, which was constructed via Student’s T test and one-way analysis of variance (ANOVA), and Tukey’s test was performed to proceed multiple variance correction on samples. The difference between the experimental groups was considered significant when *P* < 0.05.

## 3 Results

### 3.1 LncGAS5 is associated with the history of diabetes and hypertension in AMI patients

**Figure 1.**
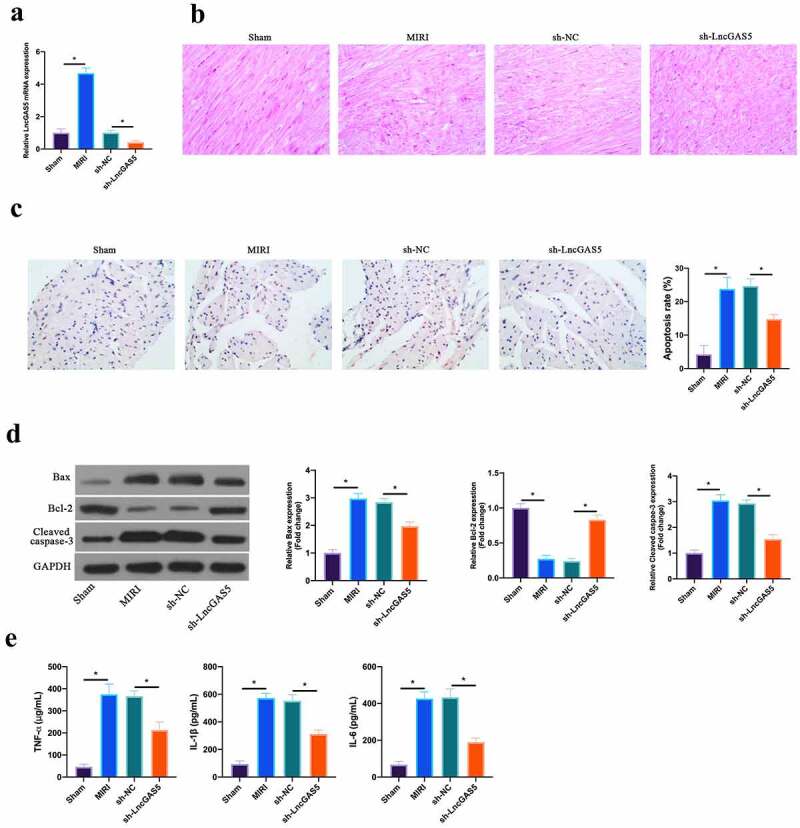
**Expression of lncGAS5 in clinical samples**.LncGAS5 expression in the blood of AMI patients before and after PCI was detected by RT-qPCR; one-way ANOVA was applied to calculate the significance of each group; the variance was corrected via Tukey’s test. *P < 0.05

In order to explore the relationship between lncGAS5 and MIRI, lncGAS5 expression after PCI was examined by RT-qPCR. As shown in Figure 1, lncGAS5 expression was clearly elevated after PCI. Subsequently, the patients were divided into the high lncGAS5 group and the low lncGAS5 group according to the median expression level of lncGAS5, and the correlation between lncGAS5 and the clinicopathological characteristics of AMI patients was examined by Spearman correlation analysis. As shown in Table 2, overexpression of lncGAS5 were associated with diabetes and hypertension in AMI patients.

**Table 2.** Correlation analysis of lncGAS5 with clinicopathological characteristics in AMI patients

Features	Groups	*n*	LncGAS5 expression		*P*
			High expression group (*n* = 13)	Low expression group (*n* = 13)	
Age	<55 years	11	4	7	0.2337
	≥55 years	15	9	6	
Gender	Male	17	7	10	0.2162
	Female	9	6	3	
Smoke	Yes	12	7	5	0.4313
	No	14	6	8	
Drink	Yes	7	2	5	0.1847
	No	19	11	8	
Diabetes	Yes	16	12	4	0.0013
	No	10	1	9	
Hypertension	Yes	12	10	2	0.0016
	No	14	3	11

### 3.2 LncGAS5 is up-regulated in MIRI; silencing LncGAS5 inhibits MIRI.

**Figure 2.**
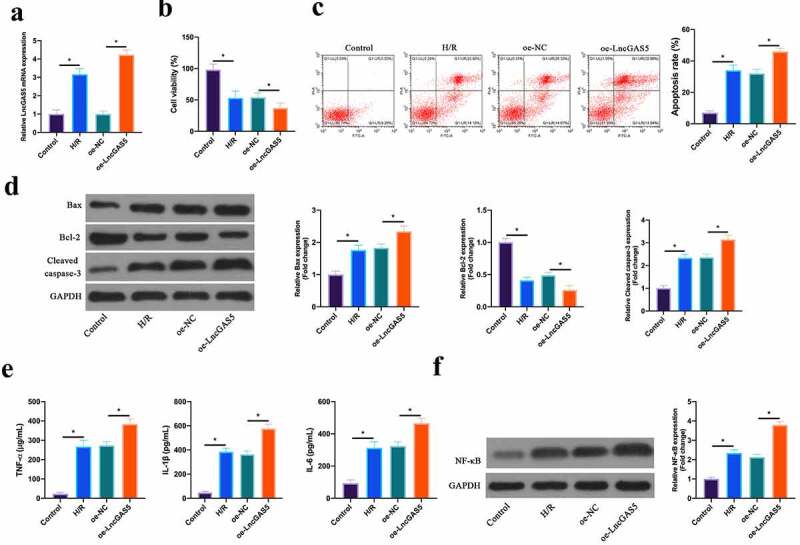
**LncGAS5 is upregulated in MIRI and silencing LncGAS5 inhibits MIRI**.A. RT-qPCR to detect LncGAS5 expression in mice myocardial tissues in the Sham, the MIRI, the sh-NC, and the sh-LncGAS5 groups; B. HE staining representative images of in mice myocardial tissues in the Sham, the MIRI, the sh-NC, and the sh-LncGAS5 groups; C. TUNEL staining to in mice myocardial tissue apoptosis in the Sham, the MIRI, the sh-NC, and the sh-LncGAS5 groups; D. Western blot to check Bax, Bcl-2 and lysed caspase-3 expressions in mice myocardial tissues in the Sham, the MIRI, the sh-NC, and the sh-LncGAS5 groups; E. ELISA to examine TNF-α, IL-1β and IL-6 levels in mice myocardial tissues in the Sham, the MIRI, the sh-NC, and the sh-LncGAS5 groups; the value was expressed as mean ± SD (*n* = 3); one-way ANOVA was applied to calculate the significance of each group; the variance was corrected via Tukey’s test. *P < 0.05

In reference to previous studies, LncGAS5 is up-regulated in MIRI model [13]. In this work, it was further confirmed, and later, through adenovirus injection, silenced LncGAS5 expression was present in MIRI model mice (Figure 2a). HE staining revealed that ischemia-reperfusion elevated myocardial tissue injury. After silencing LncGAS5, myocardial tissue injury was restored (Figure 2b). In accordance with TUNEL staining, after ischemia-reperfusion, mice cardiomyocyte apoptotic rate was elevated apparently, while after silencing LncGAS5, it was decreased clearly (Figure 2c). Additionally, Bax was promoted, but Bcl-2 expression was declined and lysed Caspase-3 protein expression was present via ischemia-reperfusion in myocardial tissues. After silencing LncGAS5, in MIRI mice myocardial tissues, Bax, lysed Caspase-3 and Bcl-2 expression was reversed (Figure 2d). Subsequently, the impact of silencing LncGAS5 on myocardial inflammation in MIRI mice was examined. IL-1β, IL-6 and TNF-α levels were facilitated via Ischemia-reperfusion in mice myocardial tissues, whereas silencing LncGAS5 observably reduced inflammatory factor levels (Figure 2e). This indicates that LncGAS5 was highly expressed in MIRI, and silencing LncGAS5 effectively protected myocardial injury resulting from ischemia-reperfusion.

### 3.3 Overexpressing LncGAS5 promotes MIRI.

**Figure 3.**
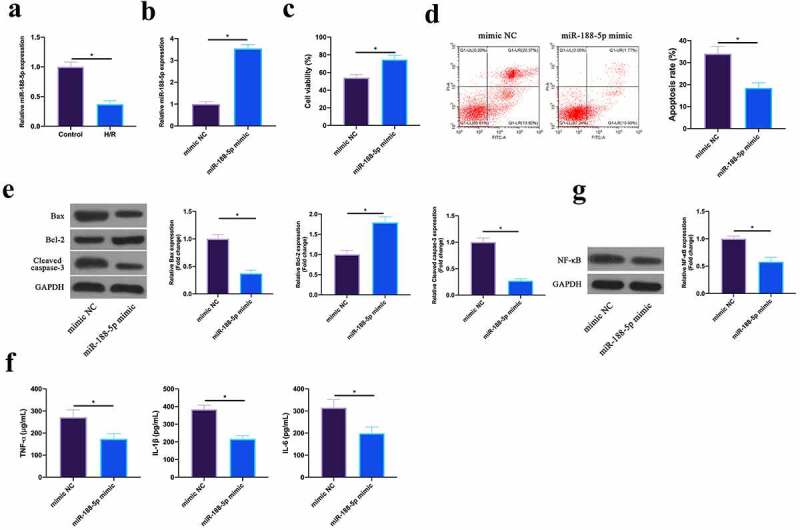
**Overexpressing LncGAS5 promotes MIRI**.A. RT-qPCR to detect LncGAS5 expression in HL-1 cells in the Control, the H/R, the oe-NC, the oe-LncGAS5 groups; B. CCK-8 to check HL-1 cell viability in the Control, the H/R, the oe-NC, the oe-LncGAS5 groups; C. Flow cytometry to check HL-1 cell apoptosis in the Control, the H/R, the oe-NC, the oe-LncGAS5 groups; D. Western blot to check Bax, Bcl-2 and lysed caspase-3 expressions in HL-1 cells in the Control, the H/R, the oe-NC, the oe-LncGAS5 groups; E. ELISA to examine TNF-α, IL-1β, and IL-6 levels in HL-1 cells in the Control, the H/R, the oe-NC, the oe-LncGAS5 groups; F. Western blot to examine NF-κB p65 expression in HL-1 cells in the Control, the H/R, the oe-NC, the oe-LncGAS5 groups; the value was expressed as mean ± SD (*n* = 3); one-way ANOVA was applied to calculate the significance of each group; the variance was corrected via Tukey’s test. *P < 0.05

Next, the impact of LncGAS5 on MIRI was further examined through *in vitro* experiments. After H/R treatment, LncGAS5 level in HL-1 cells was up-regulated clearly, and after transfecting with oe-LncGAS5, LncGAS5 level was further elevated (Figure 3a). Based on CCK-8 results, HL-1 cell viability was obviously reduced via H/R treatment, whereas the effect was further strengthened via overexpressing LncGAS5 (Figure 3b). Flow cytometry findings revealed that after H/R treatment or overexpressing LncGAS5, HL-1 cell apoptosis rate was visually increased (Figure 3c). In addition, Bax and lysed caspase-3 expression was advanced, while Bcl-2 expression was decreased in HL-1 cells via H/R treatment, while overexpressing LncGAS5 further increased or decreased Bax, lysed caspase-3 and Bcl-2 expression (Figure 3d). In terms of ELISA and western blot findings, TNF-α, IL-1β and IL-6 levels as well as NF-κB p65 expression were observably promoted via H/R treatment in HL-1 cells, while overexpressing LncGAS5 further enhanced HL-1 cell inflammation (Figure 3e, f).

### 3.4 Up-regulating miR-188-5p represses MIRI

**Figure 4.**
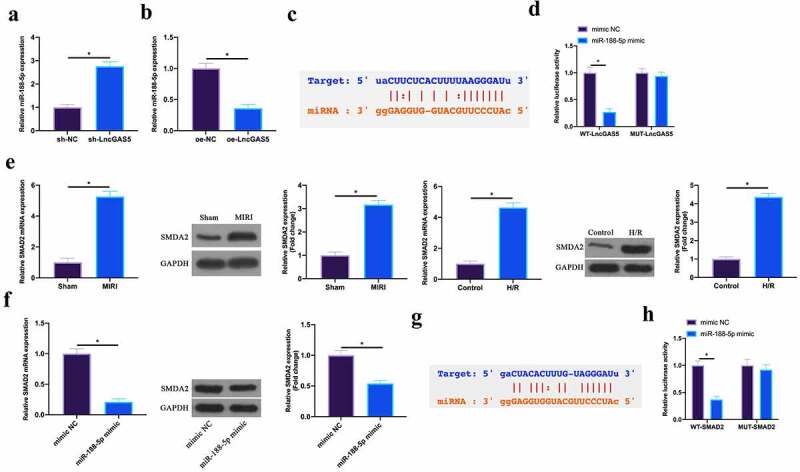
**Upregulating miR-188-5p inhibits MIRI**.A. RT-qPCR to detect LncGAS5 expression in HL-1 cells in the Control and the H/R groups; B. RT-qPCR to detect LncGAS5 expression in HL-1 cells in the mimic NC and the miR-188-5p mimic groups; C. CCK-8 to check HL-1 cell viability in the mimic NC and the miR-188-5p mimic groups; the si-NC and the si-SMAD2 groups; D. Flow cytometry to check HL-1 cell apoptosis in the mimic NC and the miR-188-5p mimic groups; E. Western blot to check Bax, Bcl-2 and lysed caspase-3 expressions in HL-1 cells in the mimic NC and the miR-188-5p mimic groups; F. ELISA to examine TNF-α, IL-1β, and IL-6 levels in HL-1 cells in the mimic NC and the miR-188-5p mimic groups; G. Western blot to examine NF-κB p65 expression in HL-1 cells in the mimic NC and the miR-188-5p mimic groups; the value was expressed as mean ± SD (*n* = 3); one-way ANOVA was applied to calculate the significance of each group; the variance was corrected via Tukey’s test. *P < 0.05

MiR-188-5p has been shown to be involved in regulating cardiomyocyte apoptosis after I/R [18]. In this work, it was found that miR-188-5p was under-expressed in HL-1 cells after H/R (Figure 4a). Subsequently, miR-188-5p expression was up-regulated via transfecting miR-188-5p mimic in HL-1 cells after H/R (Figure 4b). The findings revealed that after up-regulating miR-188-5p, HL-1 cell viability in H/R group and Bcl-2 expression were visually elevated, while the apoptotic rate, and Bax and lysed caspase-3 expression were signally reduced (Figure 4c-e). Additionally, TNF-α, IL-1β, and IL-6 levels as well as NF-κB p65 expression were reduced via up-regulating miR-188-5p in HL-1 cells (figure 4f, g). This indicated that miR-188-5p was under-expressed in H/R-treated cardiomyocytes, and upregulating miR-188-5p inhibited MIRI.

### 3.5 LncGAS5 acts as a sponge of miR-188-5p to regulate SMAD2 expression

**Figure 5.**
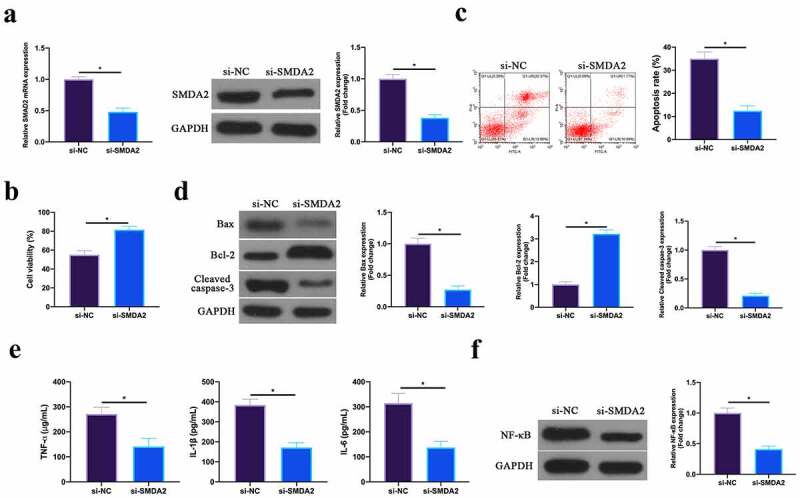
**LncGAS5 acts as a sponge of miR-188-5p to regulate SMAD2 expression**.A. RT-qPCR to detect miR-188-5p expression in mice cardiomyocytes in the sh-NC and the sh-LncGAS5 groups; B. RT-qPCR to detect miR-188-5p expression in HL-1 cells in the oe-NC and the oe-LncGAS5 groups; C. Bioinformatics website http.//starbase.sysu.edu.cn/ to predict the potential-binding sites of LncGAS5 and miR-188-5p; D. Dual luciferase reporter experiment to check targeting relationship between LncGAS5 and miR-188-5p; E. RT-qPCR and western blot to detect SMAD2 expression in mice myocardial tissue in the Sham and the MIRI groups as well as in HL-1 cells in the Control and the H/R groups; F. RT-qPCR and western blot to detect SMAD2 expression in HL-1 cells in the mimic NC and the miR-188-5p mimic groups; G. Bioinformatics website http://starbase.sysu.edu.cn/ to predict potential-binding sites of SMAD2 and miR-188-5p; H. Dual luciferase reporter experiment to check targeting relationship between SMAD2 and miR-188-5p; the value was expressed as mean ± SD (n = 3); one-way ANOVA was applied to calculate the significance of each group; the variance was corrected via Tukey’s test. *P < 0.05

In this work, the author found that miR-188-5p expression was promoted via silencing LncGAS5 in MIRI mice, while declined via overexpressing LncGAS5 in H/R cardiomyocytes (Figure 5a, b). Therefore, the author speculated that LncGAS5 might target miR-188-5p expression in MIRI. The bioinformatics website http://starbase.sysu.edu.cn/ predicted that there were potential-binding sites for LncGAS5 and miR-188-5p (Figure 5c). Subsequently, via dual luciferase reporter assay this finding was further verified. The luciferase level was obviously reduced via WT LncGAS5 in miR-188-5p mimic group, while MUT LncGAS5 had no obvious impact on the luciferase level in miR-188-5p mimic group (Figure 5d). This manifested that LncGAS5 performed targeted regulation on miR-188-5p expression.

Next, the target genes of miR-188-5p were further explored. SMAD2 has been proved to act importantly in MIRI or myocardial infarction [19,20]. Here, the author found that SMAD2 was highly expressed in MIRI mice and H/R-treated cardiomyocytes (Figure 5e). Additionally, overexpressing miR-188-5p was available to suppress SMDA2 expression (figure 5f). Therefore, the author speculated that SMAD2 was the target gene of miR-188-5p. To verify this idea, via bioinformatics website http://starbase.sysu.edu.cn/, the results found that SMAD2 and miR-188-5p had potential binding sites (Figure 5g). Further dual-luciferase reporter experiments conveyed that the luciferase activity was reduced via WT SMAD2 in miR-188-5p mimic group, while MUT SMAD2 had no impact on luciferase activity (Figure 5h). This manifested that SMAD2 was the target gene of miR-188-5p.

### 3.6 Silencing SMAD2 represses MIRI

**Figure 6.**
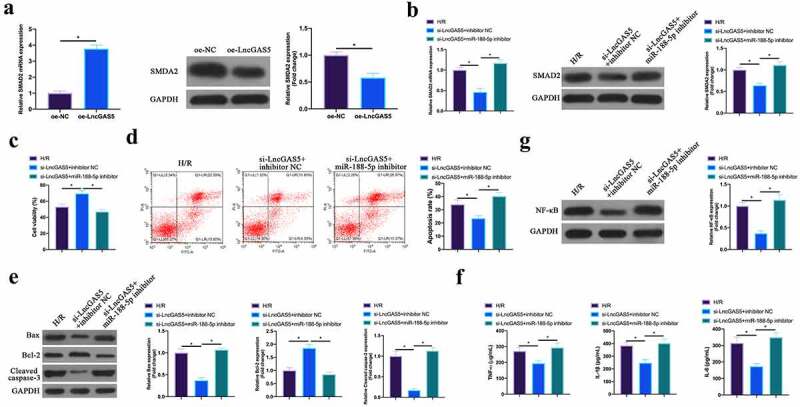
**Silencing SMAD2 inhibits MIRI**.A. RT-qPCR and western blot to detect SMAD2 expression in HL-1 cells in the si-NC and the si-SMAD2 groups; B. CCK-8 to check HL-1 cell viability in the si-NC and the si-SMAD2 groups; C. Flow cytometry to check HL-1 cell apoptosis in the si-NC and the si-SMAD2 groups; D. Western blot to check Bax, Bcl-2 and lysed caspase-3 expression in HL-1 cells in the si-NC and the si-SMAD2 groups; E. ELISA to examine TNF-α, IL-1β and IL-6 levels in HL-1 cells in the si-NC and the si-SMAD2 groups; F. Western blot to examine NF-κB p65 expression in HL-1 cells in the si-NC and the si-SMAD2 groups; the value was expressed as mean ± SD (*n* = 3); one-way ANOVA was applied to calculate the significance of each group; the variance was corrected via Tukey’s test. *P < 0.05

Next, the role of SMAD2 was examined in MIRI. SMAD2 in HL-1 cells was silenced through plasmid transfection (Figure 6a). In terms of the findings, after silencing SMAD2, HL-1 cell viability in H/R group and Bcl-2 expression were obviously elevated, while apoptotic rate, and Bax and lysed caspase-3 expression were signally reduced (Figure 6b-d). Additionally, TNF-α, IL-1β, and IL-6 levels and NF-κB p65 expression in HL-1 cells were reduced via silencing SMAD2 (Figure 6e, f). This revealed that silencing SMAD2 repressed MIRI.

### 3.7 Overexpression of SMAD2 promotes MIRI

**Figure 7.**
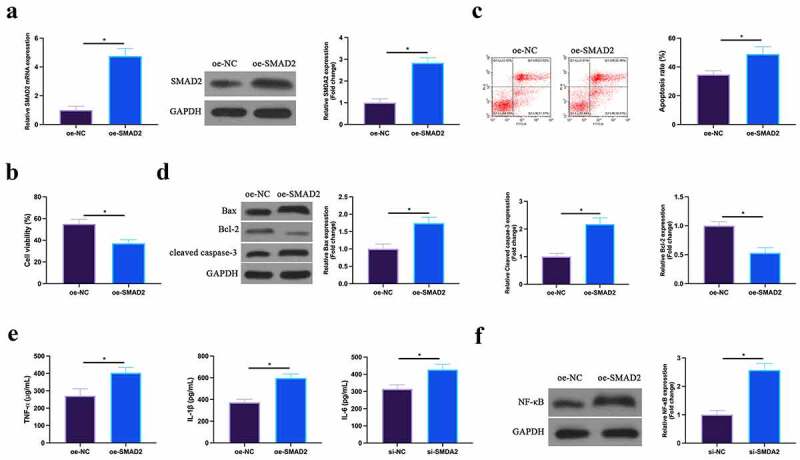
**Up-regulated SMAD2 facilitates MIRI**A. SMAD2 expression in HL-1 cells of the oe-NC and the oe-SMAD2 groups detected by RT-qPCR and western blot; B. HL-1 cell viability in the oe-NC and the oe-SMAD2 groups examined by CCK-8; C. The apoptosis of HL-1 cells in the oe-NC and the oe-SMAD2 groups detected via flow cytometry; D. The expression of Bax, Bcl-2 and cleaved caspase-3 in HL-1 cells of the oe-NC and the oe-SMAD2 groups examined by western blot; E. Levels of TNF-α, IL-1β and IL-6 in HL-1 cells of the oe-NC and the oe-SMAD2 groups detected by ELISA; F. The expression of NF-κB p65 in HL-1 cells of the oe-NC and the oe-SMAD2 groups examined by western blot. The value was expressed as mean ± SD (*n* = 3); one-way ANOVA was applied to calculate the significance of each group; the variance was corrected via Tukey’s test. *P < 0.05

The effect of overexpression of SMAD2 on H/R cardiomyocytes was then detected. As shown in Figure 7a, the expression of SMAD2 in HL-1 cells was upregulated by transfection of overexpressed (oe)-SMAD2. Functional tests manifested that overexpression of SmDA2 repressed HL-1 cell viability and Bcl-2 expression, and promoted apoptosis rate, Bax and cleaved Caspase-3 expression. The increase of the levels of TNF-α, IL-1β, and IL-6 and the expression of NF-κB p65 in HL-1 cells was also induced via up-regulation of SMAD2. This suggests that overexpression of SMAD2 could facilitate MIRI.

### 3.8 LncGAS5 regulates MIRI development through miR-188-5p/SMAD2 axis

**Figure 8.**
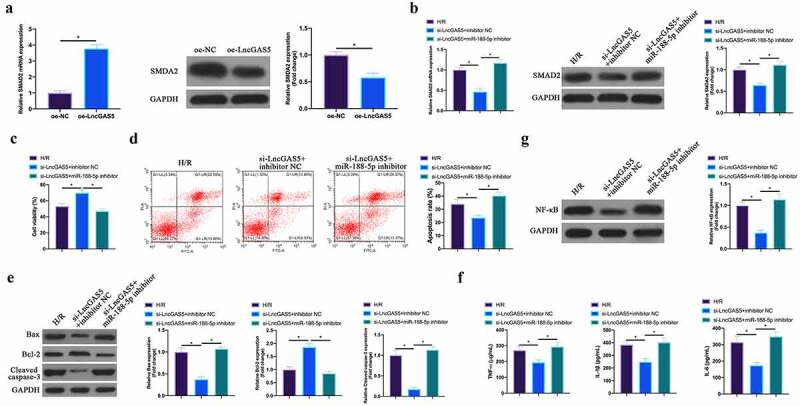
**LncGAS5 regulates MIRI development through miR-188-5p/SMAD2 axis**.A. RT-qPCR to detect SMAD2 expression in HL-1 cells in the oe-NC and the oe-LncGAS5 groups; B. RT-qPCR to detect SMDA2 expression in HL-1 cells in the H/R, the si-LncGAS5 + inhibitor NC, and the si-LncGAS5 + miR −188-5p inhibitor groups; C. CCK-8 to check HL-1 cell viability in the H/R, the si-LncGAS5 + inhibitor NC, si-LncGAS5 + miR-and 188–5p inhibitor groups; D. Flow cytometry to check HL-1 cell apoptosis in the H/R, the si-LncGAS5 + inhibitor NC, and the si-LncGAS5 + miR-188-5p inhibitor groups; E. Western blot to check Bax, Bcl-2 and lysed caspase-3 expressions in HL-1 cells in the H/R, the si- LncGAS5 + inhibitor NC, and the si-LncGAS5 + miR-188-5p inhibitor groups; F. ELISA to examine TNF-α, IL-1β and IL-6 levels in HL-1 cells in the H/R, the si-LncGAS5 + inhibitor NC, and the Si-LncGAS5 + miR-188-5p inhibitor groups; G. Western blot to examine NF-κB p65 expression in HL-1 cells in the H/R, the si-LncGAS5 + inhibitor NC, and the si-LncGAS5 + miR-188-5p inhibitor groups; the value was expressed as mean ± SD (*n* = 3); one-way ANOVA was applied to calculate the significance of each group; the variance was corrected via Tukey’s test. *P < 0.05

Next, the author probed into whether miR-188-5p/SMAD2 axis links with the process of LncGAS5 regulating MIRI. SMAD2 expression was promoted via overexpressing LncGAS5 in HL-1 cells (Figure 8a). Subsequently, si-LncGAS5 and miR-188-5p inhibitor were co-transfected into HL-1 cells. SMAD2 expression was reduced via silencing LncGAS5, while at the same time inhibiting miR-188-5p, SMAD2 expression was signally restored (Figure 8b). Additionally, after silencing LncGAS5, HL-1 cell viability was visually elevated, but apoptotic rate, Bax and lysed caspase-3 expressions were obviously reduced. TNF-α, IL-1β, and IL-6 levels and NF-κB expression in HL-1 cells were down-regulated via silencing LncGAS5. After transfecting miR-188-5p inhibitor, these impacts were visually reversed (Figure 8c-g).

## 4 Discussion

Although ischemia-reperfusion is an essential way to save AMI, the cardiomyocyte apoptosis resulting from it is still a problem that needs to be solved urgently [21,22]. In this research, the author found that silencing LncGAS5 can rescue MIRI-caused cardiomyocyte apoptosis and inflammation *in vivo* and *in vitro*. This impact is mainly due to LncGAS5 acting as a sponge targeting miR-188-5p to regulate SMAD2 expression, thereby affecting MIRI.

Bax among the most important pro-apoptotic genes in the plant, belongs to Bcl-2 family. Encoded Bax is available to repress Bcl-2 through forming a heterodimer with Blc-2 [23,24]. Additionally, caspase-3, as the most vital terminal splicing enzyme is a key gene [25] amid apoptosis. Based on a large amount of evidence, after ischemia-reperfusion, Bax and lysed caspase-3 expression was elevated while Bcl-2 expression was decreased, which is directly related to cardiomyocyte apoptosis [26,27]. Therefore, regulating apoptotic proteins expression can be a potential target for treating MIRI. Recently, LncRNA involvement in regulating MIRI cardiomyocyte apoptosis has been widely reported. For example, Yu SY et al. found that depressing lncRNA AK139328 can reduce apoptosis in MIRI mice models through regulating miR-204-3p [28]. Additionally, Han Y et al. discovered that LncGAS5 affects Bax, lysed caspase-3 and Bcl-2 expressions in H/R-treated H9c2 cells through regulating the miR-532-5p/PI3K/AKT axis, thereby promoting cell apoptosis [14]. In our work, it was found that lncGAS5 not only regulated apoptotic protein expression *in vitro*, but also obtained consistent findings in *in vivo* experiments. Additionally, lncGAS5 was also available to affect apoptotic protein expression through regulating miR-188-5p/SMAD2 axis, which indicated that lncGAS5 could regulate MIRI cardiomyocyte apoptosis through various signaling pathways.

Many evidences support that NF-κB signaling pathway is an essential target for treating ischemia-reperfusion myocardial inflammation and preventing cell apoptosis [29–31]. Recently, Yang Q et al. reported that knocking down SMAD2 protein can inhibit NF-κB signaling pathway in ischemia-reperfusion models *in vivo* and *in vitro*, thereby preventing kidney inflammation [32]. In this research, the authors found that silencing LncGAS5 or upregulating miR-188-5p effectively inhibits SMAD2 expression, which helps prevent NF-κB phosphorylation in MIRI model and reduces the pro-inflammatory cytokines TNF-α, IL-1β and IL-6 levels. For a long time, apoptosis has been regarded as a vital mechanism of host defense, especially in inflamed tissues, as a prominent pathological feature [33]. But based on recent studies, apoptosis may be a direct cause of immune response [34,35]. Therefore, LncGAS5 may reduce cell apoptosis through miR-188-5p/SMAD2 pathway, thereby regulating NF-κB inflammation signaling pathway and downstream pathways to prevent MIRI tissue inflammation along with further apoptosis. The specific mechanism needs further exploration.

In this study, it was found that there was the up-regulation of lncGAS5 expression after CPI in AMI patients, and elevation of lncGAS5 was correlated with the history of diabetes and hypertension in AMI patients. This suggests that lncGAS5 may be a promising diagnostic criterion for MIRI. However, owing to the limitations of this study, it is still unclear whether lncGAS5 can be used as a clinical therapeutic target to achieve similar effects as those in animal models or cell models.

## 5 Conclusion

To conclude, this work implies that silencing LncGAS5 is available to prevent MIRI cell apoptosis and inflammation *in vivo* and *in vitro* through regulating miR-188-5p/SMAD2 axis, thereby saving cardiomyocyte damage. LncGAS5/miR-188-5p/SMAD2 may be an essential target for treating MIRI in the future.

## Supplementary Material

Supplemental Material
