# Supplementary figures and images for: Long non-coding RNA growth arrest specific transcript 5 acting as a sponge of MicroRNA-188-5p to regulate SMAD family member 2 expression promotes myocardial ischemia-reperfusion injury
Source: Bioengineered. 2021 Oct 10;12(1):6674–86. doi: 10.1080/21655979.2021.1957524 (PMC8806717; doi:10.1080/21655979.2021.1957524)

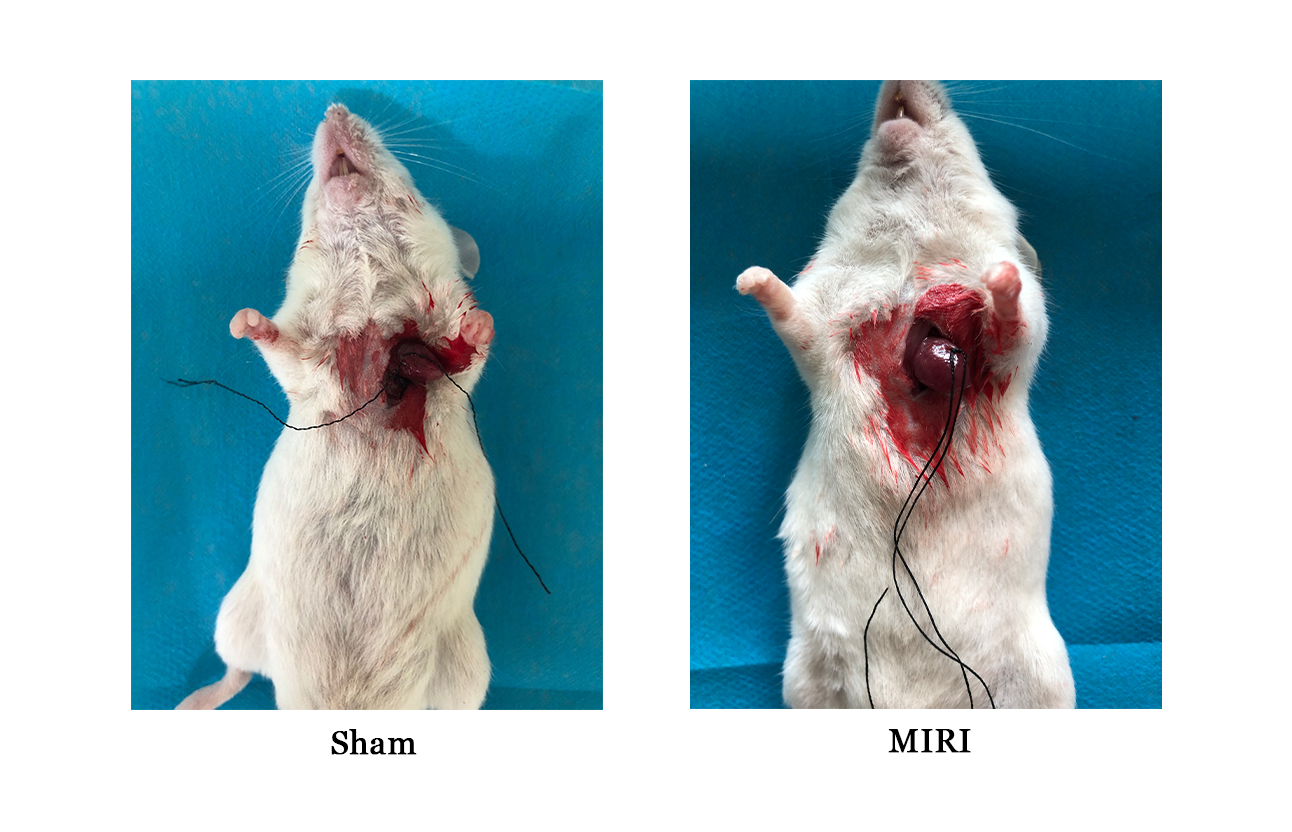

Supplement: Supplemental Material [file KBIE_A_1957524_SM9900.zip › Figure S1.tif]
